# Viral pathogen infection drives deterministic assembly of plant endophytic microbiomes: soil properties as divergent drivers and low-abundance taxa as primary responders

**DOI:** 10.3389/fmicb.2026.1866391

**Published:** 2026-06-24

**Authors:** Jin Hu, Erwei Zhao, Yi He, Xiaohui Huang, Wenyi Liu, Xiaowu Xiang, Yanping Tan, Xi Chen, Yabing Gu, Zhenghua Liu, Huaqun Yin, Qiansi Chen, Delong Meng

**Affiliations:** 1 Central South University School of Minerals Processing and Bioengineering, Changsha, China; 2 Key Laboratory of Biohydrometallurgy, Ministry of Education, Changsha, China; 3 Bijie Tobacco Corporation, Bijie, Guizhou, China; 4 Hunan Edible Fungi Institute, Changsha, China; 5 Dongkou County Agricultural Bureau, Shaoyang, China; 6 The Fourth Vocational Secondary School in Nanning City, Nanning, China; 7 Hunan Institution of Microbiology, Changsha, China; 8 China National Tobacco Corp Zhengzhou Tobacco Research Institute, Zhengzhou, China

**Keywords:** community assembly, deterministic processes, plant endophytes, soil–plant biological health, viral pathogen infection

## Abstract

Understanding how viral pathogens reshape plant endophytic community assembly is essential for assessing soil biological health, given that soil is the primary microbial reservoir, but key knowledge gaps persist. Using 16S rRNA amplicon sequencing, we investigated potato virus Y (PVY) infection effects on the assembly processes in plant endophytic community and their associations with soil properties. PVY infection significantly affected β-diversity (but not α-diversity) of endophytes in roots, but had no significant impact on either α- or β-diversity in leaves and enhanced deterministic assembly by 17% in roots and 3% in leaves. In infected plant root, endophytic community exhibited increased significantly in the importance of heterogeneous selection (HeS) and homogeneous selection (HoS) alongside decreased significantly in dispersal limitation (DL). Conversely, the importance of drift (DR) significantly increased in endophytic community assembly for infected leaves. Such virus-induced deterministic assembly was significantly correlated with soil physicochemical properties. For example, pH negatively correlated with the importance of HeS in endophytic community of infected leaves (*ρ* = −0.943), while that was reverse for infected roots (*ρ* = 0.943). Available potassium (AK) positively correlated with HeS in leaves (*ρ* = 0.986), while that was negative in roots (*ρ* = −0.899). Furthermore, PVY infection influenced a subset of low-abundance taxa (≤5%) rather than reshaping the overall community structure, including *Delftia* (bin 17) and *Allorhizobium* (bin 5), which are known contributors to soil biological health. Taken together, these findings demonstrate that viral pathogens enhance deterministic assembly in plant endophytic community, which was greatly constrained by soil properties.

## 1 Introduction

Plant endophytes are mainly recruited from soil microbial communities, and their functional integrity determines soil biological health. As part of the plant-associated microbiota, these endophytes form complex associations with plants and play important roles in promoting plant productivity and health in natural environments (Trivedi et al., 2020; Vandenkoornhuyse et al., 2015). They can promote plant growth, nutrient uptake and pathogen resistance (Yu et al., 2022). Some kinds of plant endophytes can facilitate the acquisition of resources from the environment, in addition, they can provide or regulate various plant hormones for modulate the plant growth (Santoyo et al., 2016). Under the pressure of the virus pathogen infection, plant endophytes and their metabolites can supply mineral nutrients, hormonal modulation and protection from the pathogenic organisms (Kour et al., 2024; Pieterse et al., 2014).

Pathogenic infection can alter plant phenotypes and influence the plant endophytes community. The rice blast infection changes the endophyte community in different plant compartments, and some endophytes enriched in infected plants might have roles in the defense against the pathogen (Roy et al., 2024). The endophyte bacterial community may play a crucial role in the disease process after *Ganoderma yamadae* infection on the apple leaves, through regulating their own metabolism to protect their own community of the disease (Li Y. et al., 2024). *Hymenoscyphus fraxineus* infection can disturb stable endophyte associations, and the higher infection loads can lead the stronger characterized at the leaf microbial network than the lower infection levels (Griffiths et al., 2020). The pathogen infection can make the significant differences in bacterial diversity and community composition in *Anoectochilus roxburghii*, and the pathogen migrate from soils to roots and finally the stems (Xing et al., 2022). The pathogenic virus can change the endophytes community, and the effects on plants is related to the species of the virus and the plants. While the impacts of fungal pathogens on the dynamics of plant endophytic communities have been extensively documented, the effects of viral infections, in contrast, remain comparatively less explored, especially those caused by RNA viruses.

Potato virus Y (PVY, family Potyviridae, genus *Potyvirus*) is a positive single stranded RNA virus (Hu et al., 2009; Rybicki, 2015). The PVY genome is approximately 9.7 kb in length. The RNA genome has two non-translated regions(NTRs) including 5′-NTR and 3′-NTR, which bordering a single open reading frame(ORF) that encodes for a large polyprotein which is processed by proteases P1, HC-Pro, and NIa-Pro, to 10 mature viral proteins (Chikh-Ali and Karasev, 2023; Quenouille et al., 2013). PVY virus is a major pathogen of potato, peppers and solanaceous vegetable crops (Morel et al., 2000; Scholthof et al., 2011). The symptoms of PVY virus vary depending on the virus strain, crop type, and environmental conditions. The PVY infection can result in the leaf malformation and vein necrosis (Torrance and Talianksy, 2020). However, the current understanding of PVY largely remains at the phenomenological level, while its influence on the ecological assembly rules of the plant microbiome remains largely unexplored.

Plant endophytic microbiome assembly is shaped by multiple biotic and abiotic factors, including plant compartment, host genetics, climate, and soil types (Chen et al., 2018; Xu et al., 2021; Zhang et al., 2022). Pathogen invasion is one of the major biotic stresses affecting plant microbiome assembly except for the host selection and herbivory (Fernandez-Gonzalez et al., 2020). Previous studies have demonstrated that the rhizosphere is a critical compartment of the plant, and its microbiome is closely related to plant performance (de Vries et al., 2020). On the other hand, the phyllosphere microbiome may play essential roles in plant heath and ecosystem function (Laforest-Lapointe et al., 2017).

To date, however, most related studies only focused on the rhizosphere or phyllosphere microbiomes, and a systematic understanding of microbiome structure, functions and microbial assembly in plant compartments including roots, and leaves under pathogen invasion, as well as the correlation between soil physicochemical properties and microbial assembly remains unclear. Also, the microbial community assembly is influenced by the cooperative and competitive interactions among the massively microbial members that perform functions for plant health as a whole (Duran et al., 2018; van der Heijden et al., 2016). Therefore, the succession and assembly of microbial communities should be explored to improve that how disease-induced changes the microbial community and microbial assembly in different compartments of plants.

Critically, the vast majority of existing studies have concentrated on bacterial or fungal pathogens. In contrast, how viral pathogens—particularly RNA viruses such as Potato virus Y (PVY)—reshape the assembly processes of plant endophytic communities remains almost entirely unknown. This knowledge gap severely limits our understanding of plant–microbe interactions under viral disease pressure and hinders the development of microbiome-based strategies for sustainable disease management.

To address this gap, we need a theoretical framework for quantifying community assembly processes. In traditional niche theory, deterministic factors shape microbial community structure, microbial community competition, and cooperation. Niche differentiation explains how microbial community evolution relates to environmental factors (Stegen et al., 2013), Selection arises from biotic and abiotic pressures that cause variation in reproductive success across individuals and species. Dispersal determines the extent to which individuals move between communities. Drift results from random fluctuations in population sizes. Speciation can drive differences in species richness among communities that are isolated from dispersal (Vellend, 2010). Turnover in community composition within a meta-community is therefore governed by a combination of selection, dispersal and drift. Quantifying the relative importance of stochastic versus deterministic processes can thus elucidate the mechanisms of microbial community assembly. Several approaches, such as phylogenetic null models and the neutral community model (NCM), have been developed for this purpose.

In natural systems, viruses are recognized as important approach for regulating microbial community. For example, with the process of *Pectobacterium atrosepticum* infection in potato tubers, the endophytes community has constant change (Koiv et al., 2015). The *Ralstonia solanacearum* infection to tobacco can change microbial composition and reduce the endophytes community diversity in root and stem (Tao et al., 2024). However, whether viral pathogen infection similarly alters the assembly processes of plant endophytic microbiomes remains poorly understood.

This study aims to investigate whether viral pathogen infection alters the assembly processes of the plant endophytic microbiome and to quantify the relative contributions of deterministic versus stochastic processes under viral stress in the context of soil–plant biological health. By comparing the endophytic bacterial communities in different tissues (roots and leaves) of healthy and Potato virus Y (PVY)-infected plants, we employed high-throughput amplicon sequencing and quantitative analyses based on phylogenetic null modeling and the neutral community model. Therefore, we hypothesize that viral infection in plants leads to a more deterministic assembly of the endophytic community, further proposing that PVY infection primarily influences a subset of low-abundance taxa (≤5%) rather than reshaping the overall community structure. We also investigate how soil physicochemical properties (pH, nitrogen, potassium, water content, and organic matter)—key indicators of soil biological health—correlate with the assembly processes. Based on our empirical data, we find that these soil factors exhibit significant correlations with deterministic processes (e.g., homogeneous or heterogeneous selection), with roots and leaves showing opposite response patterns to the same soil factor (e.g., pH and potassium). By elucidating how a viral pathogen restructures endophytic assembly and how soil properties differentially regulate this process in roots versus leaves, this study provides mechanistic insights into the disruption of plant–microbiome interactions under disease stress. Our findings offer a theoretical foundation for promoting sustainable agricultural practices through improved management of soil biological health, particularly in virus-prone agroecosystems.

## 2 Materials and methods

### 2.1 Sampling

All samples were collected from fields of tobacco (*Nicotiana tabacum* L.) cultivar “Yunyan 87” located in China (29°13′30″–29°59′19″N and 110°28′40″–110°58′30″E). This region has experienced one of the most severe disease outbreaks on local tobacco in recent years, and PVY isolates from this region predominantly induce typical venous necrosis and pronounced stunting symptoms. Besides, the soil was classified as red soil (according to the Chinese Soil Taxonomy) originating from typical tobacco-growing fields in Hunan. Plant tissues (roots and leaves) and rhizosphere soils were sampled in August 2022.

Plants were grouped based on typical field symptoms using traditional visual assessment practices widely adopted in plant pathology. Tobacco plants exhibiting significant height reduction and veinal necrosis were classified as PVY-infected, whereas those showing no obvious symptoms were classified as healthy (Chin et al., 2020; Latorre, 1984). Six replicates were collected for each of the healthy and PVY-infected groups. All samples were transported to the laboratory on dry ice and stored at −80 °C until further experiment (Cao et al., 2024; Xiong et al., 2023). Notably, all fields in this study were planted with the same tobacco cultivar and managed under identical agronomic practices. Therefore, any potential confounding effects arising from management or genetic background were uniformly distributed between the symptomatic and asymptomatic groups, which supports the validity of comparative analyses.

Tissues were excised from leaves showing typical veinal necrosis and from healthy plants, then thoroughly ground in a buffer solution at a weight-to-volume ratio of 1:20. After grinding, the top of the mesh sample bag was cut open, and the ground sample was placed between the mesh linings of the bag. The bag was repeatedly crushed from the outside to ensure complete disruption and homogenization. The PVY visual strip assay was removed from its packaging and inserted into the ground buffer solution (Xu et al., 2023).

This study employed a two-factorial design, with plant health status (healthy vs. PVY-infected) and tissue type (root vs. leaf) as the two factors. This approach thus resulted in four sample types: healthy roots (HR), PVY-infected roots (IR), healthy leaves (HL), and PVY-infected leaves (IL).

Plant tissues (about 5 g) were successively immersed in 70% ethanol for 10 min, 5.25% sodium hypochlorite solution for 5 min, and 70% ethanol for 1 min, and finally washed with sterile water (Gao et al., 2021; Liu et al., 2021). Treated tissues were ground with liquid nitrogen in a sterile mortar and then stored at −80 °C for further microbial experiments.

### 2.2 DNA extraction and amplicon sequencing

Total DNA was extracted from plant samples using the FastDNA SPIN Kit for Soil, following the manufacturer’s instructions. The primer 799F (5′-AACMGGATTAGATACC CKG-3′)/1115R (5′-AGGGTTGCGCTCGTTG-3′) was used to amplify the V5–V7 region of the bacterial 16S rRNA gene (Deng et al., 2021; Kembel et al., 2014; Xiong et al., 2021). Sequencing was performed using the Illumina Hiseq2500 platform at MEGIGENE Biotechnology Co., Ltd. (Guangzhou, China). Based on the manufacturer’s instructions (Illumina), the genomic DNA was fragmented to approximately 300 bp using a Covaris M220 sonicator. A paired-end sequencing library was constructed with the TruSeq™ DNA Sample Prep Kit, which involved ligating Y-shaped adapters, removing self-ligated adapter fragments via magnetic bead purification, and enriching the library by PCR amplification. Bridge PCR amplification was subsequently performed using the HiSeq 3000/4000 PE Cluster Kit to generate clonal DNA clusters on the flow cell. Finally, paired-end sequencing (2 × 150 bp) was carried out on the Illumina HiSeq platform (HiSeq 3000/4000 SBS Kits), wherein fluorescently labeled nucleotides were incorporated and imaged in each cycle to determine the nucleotide sequence. The 16S rRNA sequences were processed using the QIIME2 platform (2022.8) with default parameters (Zhang et al., 2022). First, primers from reads were removed to obtain clean sequences. Then, DADA2 was used to generate feature tables based on the clean sequences (Callahan et al., 2016). Finally, the taxonomic assignment was conducted according to the SILVA reference database (SILVA SSU rRNA database release 138) for bacteria (Quast et al., 2013). Singlet reads and bacterial amplicon sequence variants (ASVs) classified as chloroplast, mitochondrion, or Viridiplantae were removed. The raw sequencing data has been deposited in the NCBI database under BioProject accession number PRJNA946037.

### 2.3 Soil physicochemical

Soil pH was measured using a pH meter in a 1:2.5 (w/v) soil–water suspension (Zhou et al., 2017). Water content was determined by oven-drying at 105 °C to constant weight. Organic matter was measured using the Walkley–Black potassium dichromate oxidation method. Total nitrogen was determined by the Kjeldahl digestion method (Zhang et al., 2019). Alkali-hydrolyzable nitrogen was measured using the alkaline hydrolysis diffusion method. Total phosphorus was determined by the molybdenum-antimony colorimetric method. Available phosphorus was quantified by the molybdenum-antimony colorimetric method (Murphy and Riley, 1962). Total potassium and available potassium were determined by flame photometry (Pansu and Gautheyrou, 2006). Correlations between soil physicochemical properties and microbial assembly processes were analyzed using Spearman’s rank correlation test in *R*, and *p* < 0.05 was considered statistically significant.

### 2.4 Data analysis

Alpha diversity indexes, including Shannon index, Simpson index and Chao1, were calculated using the vegan package (version 2.6.4) (Oksanen et al., 2013) in *R* (version 4.0.0). Differences in alpha diversity between healthy and infected plants were tested using Student’s *t*-test. LEfSe (Linear discriminant analysis Effect Size) analysis was performed using the microeco package in *R*, which includes non-parametric Kruskal-Wallis test followed by linear discriminant analysis (Segata et al., 2011). Based on the phylogenetic tree, the weighted UniFrac distance (*α* = 1) was used to evaluate *β*-diversity of microbial communities among different treatment groups, as implemented in the “microeco” package. Student’s *t*-test were performed to compare within-group distances between specific group pairs (HR vs. IR, HL vs. IL), with the significance level set at *α* = 0.05. Significance indicators (*p < 0.05, **p < 0.01, ***p < 0.001) were added to boxplots using the “ggpubr” package. Additionally, ANOSIM (Analysis of Similarities) based on the weighted UniFrac distance matrix was conducted with 999 permutations. ANOSIM generated an *R* statistic (*R* > 0 indicates greater between-group than within-group dissimilarity) and its associated *p*-value to evaluate overall differences in community structure among groups.

To investigate the succession of microbial community assembly in PVY-infected plants across different tissues (roots and leaves), the microbial communities were analyzed separately for each sampling location. Microbial community assembly processes were modeled using an ecological null model (permutation = 999) (Zhou et al., 2013). The microbial community assembly processes were divided into two processes including deterministic processes and stochastic processes according to the βNTI values. Furthermore, each of these processes can be further partitioned into sub-processes based on the RCbray values. In detail, the deterministic processes are divided into two sub-processes including the heterogeneous selection (HeS) and the homogeneous selection processes, while the stochastic processes are divided into three aspects: dispersal limitation, homogenizing dispersal, and drift (Wang et al., 2024; Zhang et al., 2022; Zhou et al., 2014). The assembly model of different microbial groups was investigated by the Infer Community Assembly Mechanisms by Phylogenetic-bin-based null model analysis (iCAMP) (Ning et al., 2020). In the iCAMP analysis, the following parameters are set: The phylogenetic metric used is the branch Mean Pairwise Distance (bMPD), the threshold for the standardized effect size (SES) is set to 1.96, the correlation threshold is set to 0.95, the confidence interval threshold is set to 0.975, and the significance index is set to “Confidence,” which is used to evaluate the significance of community assembly mechanisms.

## 3 Results

The PVY visual strip assay results (Supplementary Figure S1) showed that samples classified as PVY-infected based on field symptoms tested positive, as indicated by the appearance of a red test line alongside the control line. In contrast, healthy control samples tested negative, showing only the control line without a test line.

### 3.1 Microbial community composition and diversity in different parts of plants

A total of 716,213 high-quality bacterial 16S rRNA reads were obtained from 24 samples, which were sorted into 1,642 ASVs. Among these, 1,325 ASVs were detected in roots and 542 in leaves, with 225 ASVs shared between the two tissues.

The number of ASVs was higher in PVY-infected plants than in healthy plants in both roots and leaves. Specifically, PVY infection increased the ASV count from 939 to 974 in roots and from 344 to 410 in leaves.

**Figure 1:**
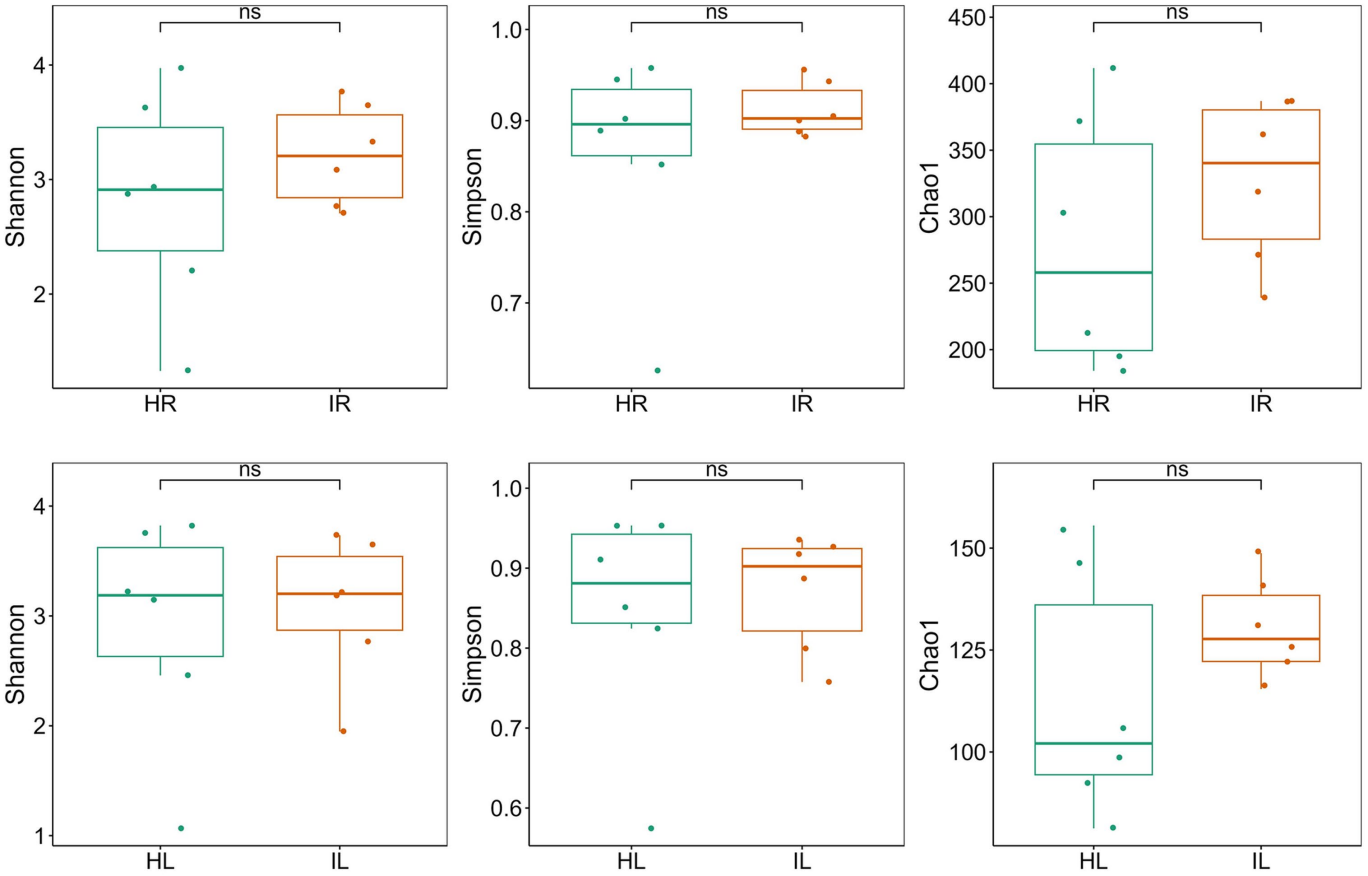
Bacterial diversity indices in different plant organs (H: healthy; I: PVY pathogen infection; R: roots; L: leaves).

As Figure 1 shows, alpha diversity indexes (Shannon index, Simpson index and Chao1), were slightly increased in PVY-infected plants. However, Student’s *t*-test revealed no significant differences (*p* > 0.05) in these indexes between PVY-infected and healthy plants, in either root or leaf endophytic communities.

**Figure 2:**
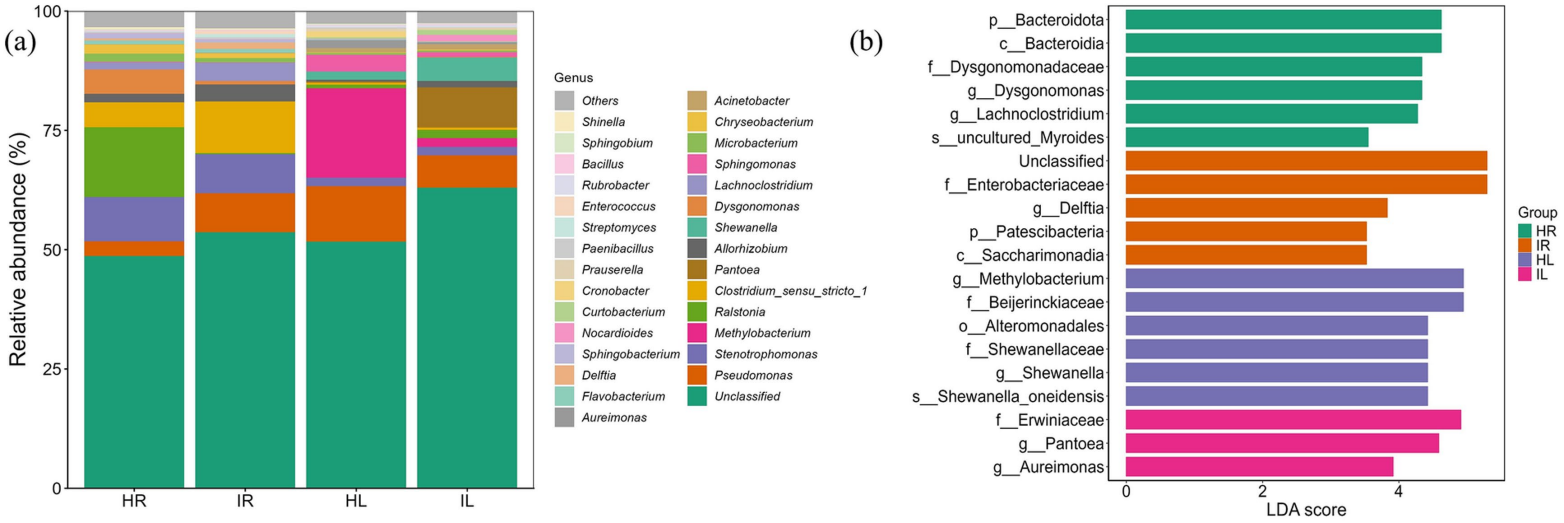
Bacterial community composition in different organs. **(a)** Relative abundances of the top 30 bacterial communities among all treatments at genus level. **(b)** Linear discriminant analysis effect size (LEfSe) analysis of top 20 microbial communities among all treatments presented on distribution histogram.

Figure 2a presents the top 30 genera in terms of relative abundance across all plant endophyte samples. In roots, PVY infection increased the relative abundance of genera such as *Pseudomonas*, *Clostridium_sensu_stricto_1*, and *Allorhizobium*, while it decreased the abundance of other major genera including *Stenotrophomonas*, *Ralstonia*, and *Dysgonomonas*. In the leaves, infection led to the enrichment of *Stenotrophomonas*, *Ralstonia*, *Pantoea*, *Allorhizobium*, and *Shewanella*, whereas genera such as *Pseudomonas* and *Methylobacterium* were reduced.

LEfSe analysis (Figure 2b) identified significant features at the genus level: *Pantoea* and *Aureimonas* were discriminative in leaves, while *Delftia* was a significant feature in roots of PVY-infected plants.

**Figure 3:**
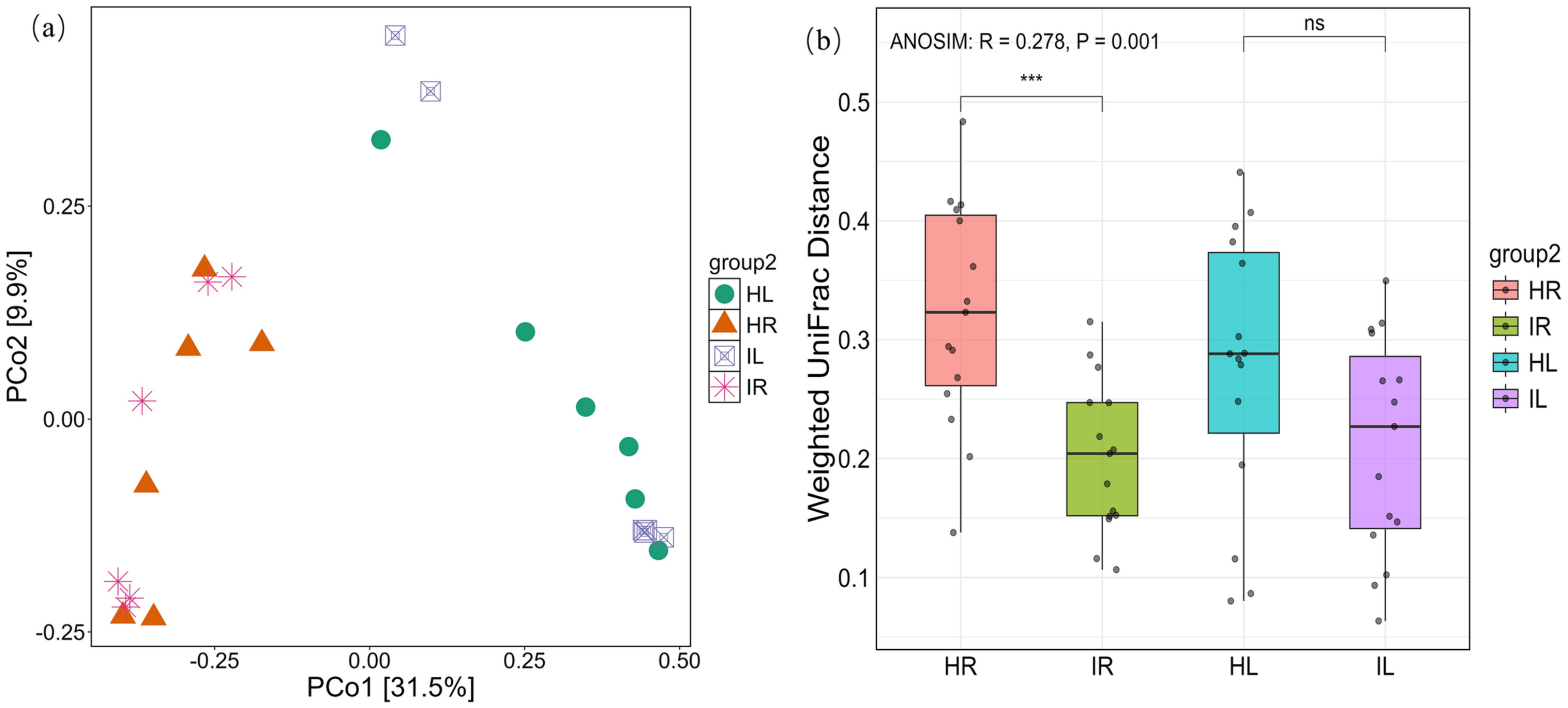
Bacterial beta diversity in the different organs. **(a)** Principal coordinates analysis (PCoA) analysis, **(b)** Weighted UniFrac distance-based clustering analysis, Statistical analysis was performed using Student’s *t*-test.

Beta diversity analysis based on the weighted UniFrac distance (Figure 3) revealed distinct responses of endophytic communities to PVY infection between roots and leaves. Within-group comparisons showed that the mean within-group weighted UniFrac distance differed significantly between healthy (HR) and infected (IR) root samples (*t*-test, *p* < 0.05), whereas no significant difference was observed between healthy (HL) and infected (IL) leaf samples (*p* > 0.05). Furthermore, ANOSIM across all four groups (HR, IR, HL, IL) indicated a statistically significant but relatively weak separation among groups (*R* = 0.278, *p* = 0.01, permutations = 999). Taken together, PVY infection significantly altered the *β*-diversity of root endophytic communities but had little effect on leaf endophytic communities, and the overall compositional differences among the four groups were modest.

### 3.2 The bacterial community assembly processes in different parts of plants

**Figure 4:**
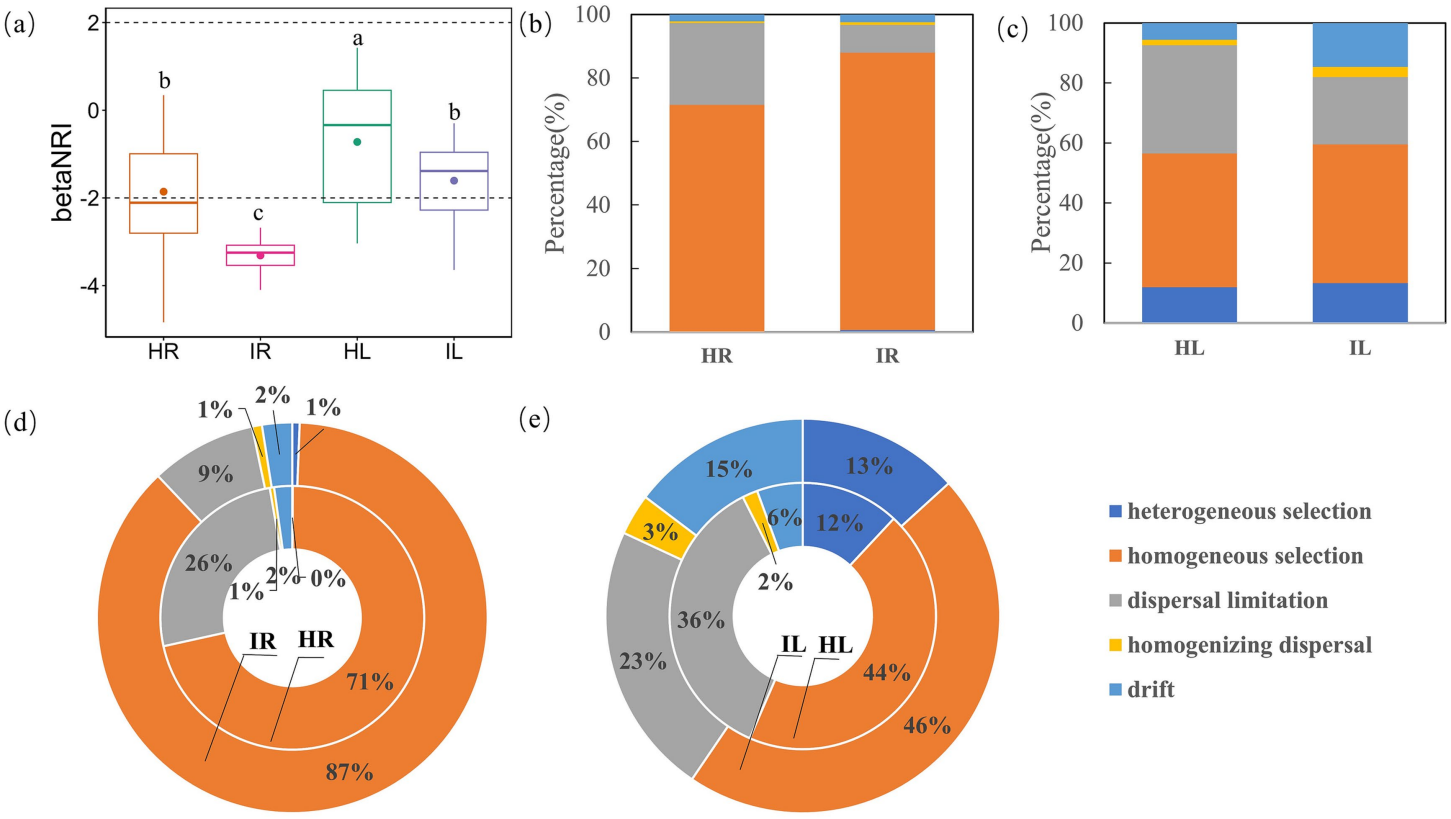
Relative importance of ecological processes inferred by iCAMP. The deterministic proportion **(a)** and assembly processes in roots **(b,d)** and leaves **(c,e)** were quantified using the null model framework implemented in iCAMP.

Figure 4a reveals that deterministic processes dominated root microbial community assembly in both healthy and PVY-infected plants, with homogeneous selection being stronger in infected plants. In contrast, stochastic processes exerted a greater influence on the leaf microbiota. However, PVY infection induced a shift in the leaf microbial community toward deterministic processes, which indicates that viral pathogen infection promotes the deterministic assembly of plant endophytes. PVY infection increased the contribution of deterministic processes to bacterial community assembly by 17% in the roots and by 3% in the leaves (Figures 4b–e).

In roots (Figure 4d), homogeneous selection and dispersal limitation were the dominant sub-processes, together accounting for over 90%. In leaves (Figure 4e), drift also played a relatively important role.

As shown in Figures 4d,e and Table 1, PVY pathogen infection significantly increased deterministic processes in root bacterial community assembly. In detail, heterogeneous selection (HeS) increased by 1% (*p* = 0.024), the homogeneous selection (HoS) showed a more pronounced 16% increase (*p* = 0.006). Meanwhile, PVY pathogen infection significantly reduced the contribution of dispersal limitation (DL), a stochastic process (*p* = 0.005), whereas, other stochastic sub-processes including homogenizing dispersal (HD) and drift (DR) increased but not significantly (*p* = 0.145 in HD, *p* = 0.576 in DR).

**Table 1:** Statistical analysis on effects of PVY infection on community assembly in different plant organs.

Organs	R					L				
Process	HeS	HoS	HD	DL	DR	HeS	HoS	HD	DL	DR
*p*-value (H vs. I)	0.024	0.006	0.145	0.005	0.576	0.851	0.815	0.251	0.185	0.048

Results are *p*-values based on *T*-test. HeS, heterogeneous selection; HoS, homogeneous selection; HD, homogenizing dispersal; DL, dispersal limitation; DR, drift; R, root; L, leaf.

In leaves, the deterministic processes (heterogeneous selection and homogeneous selection) showed higher relative importance under the pressure of PVY pathogen infection, although the increases were not significant (*p* = 0.851 for HeS, *p* = 0.185 for HoS). However, the contribution of DR, a stochastic processes, significantly increased under PVY pressure in leaves (*p* = 0.048).

### 3.3 Associations of soil physicochemical properties with assembly processes

**Figure 5:**
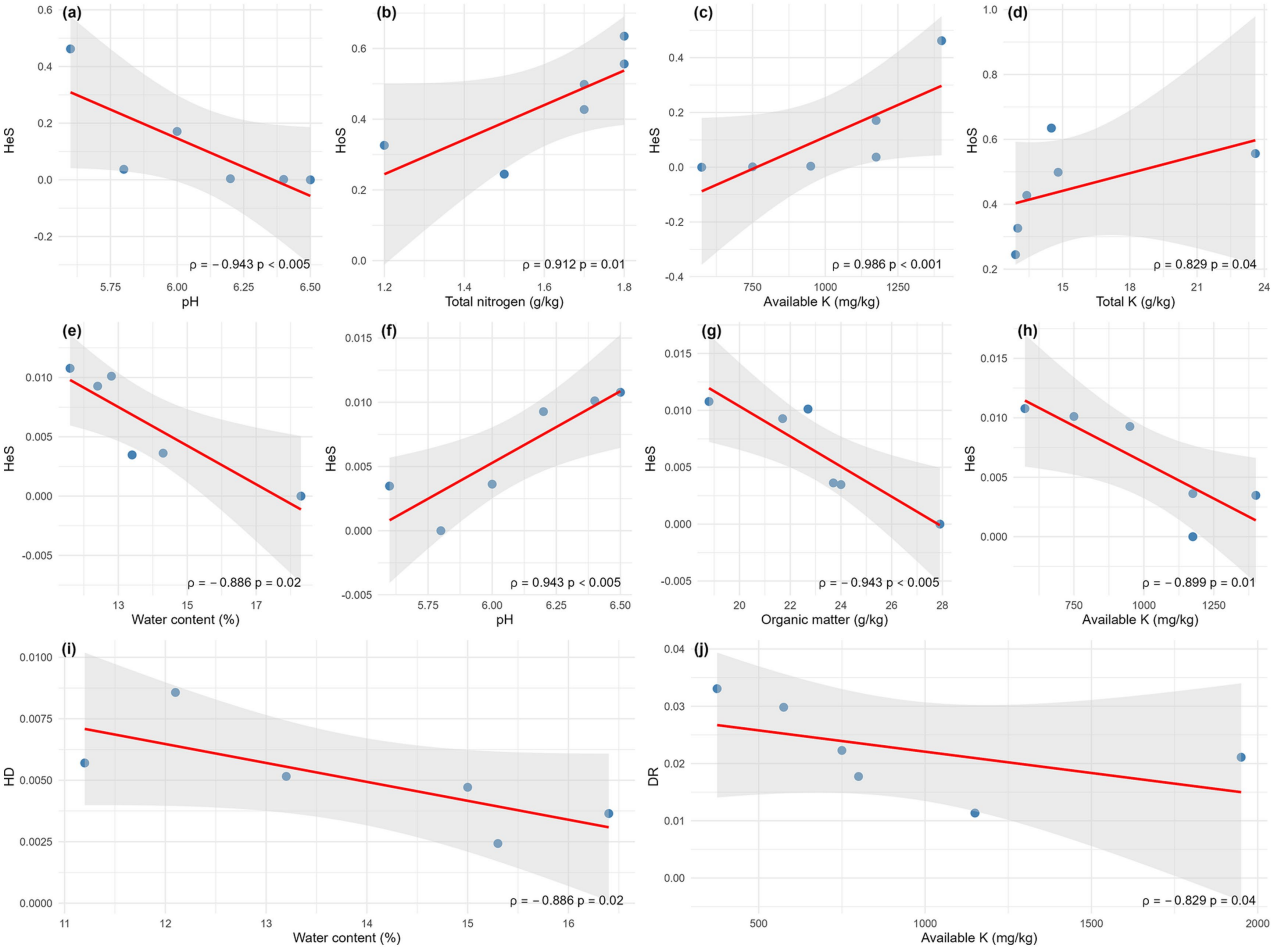
Spearman correlation analysis between soil physicochemical properties and endophytic assembly processes (significant results only, *p* < 0.05). **(a–d)** Infected leaves; **(e–h)** infected roots; **(i,j)** healthy roots.

Correlation analysis (Figure 5) revealed that, in PVY-infected plants, the ecological processes of endophytic microbial communities in leaves and roots showed significant correlations with multiple soil physicochemical properties (*p* < 0.05), and the response patterns to soil factors were markedly different between the two tissues.

As shown in Figure 5a–d, in infected leaves, the proportion of heterogeneous selection (HeS) exhibited a strong negative correlation with pH (*ρ* = −0.943) and positive correlation with available potassium (AK) (*ρ* = 0.986), indicating that lower pH conditions and higher available potassium content enhance heterogeneous selection. Meanwhile, the proportion of homogeneous selection (HoS) was significantly positively correlated with total nitrogen (TN) and total potassium (TK) (*ρ* = 0.912 and 0.829, respectively), suggesting that enrichment of nitrogen and potassium promotes the homogeneous assembly of endophytic communities in infected leaves.

In contrast, in the roots of infected plants (Figures 5e–h), heterogeneous selection (HeS) showed an opposite driving pattern: it was strongly positively correlated with pH (*ρ* = 0.943), but significantly negatively correlated with water content, organic matter (OM), and available potassium (AK) (*ρ* = −0.886, −0.943, and −0.899, respectively), indicating that under conditions of higher moisture, higher organic matter, or higher available potassium, the role of heterogeneous selection in root endophytes is weakened.

In healthy plants, no significant correlations were detected between leaf endophitic ecological processes and any of the measured soil properties (*p* > 0.05). However, two significant correlations were found in healthy roots (Figures 5i,j): the proportion of homogenizing dispersal (HD) showed a strong negative correlation with soil water content (*ρ* = −0.886), and the proportion of drift (DR) showed a moderate negative correlation with available potassium (*ρ* = −0.829).

Overall, infection status significantly altered the regulation of endophytic community assembly by soil physicochemical factors. Notably, between the leaves and roots of virus-infected plants, the effects of the same soil factors (e.g., pH and available potassium) on heterogeneous selection were completely opposite, revealing a differential response mechanism of root and leaf microenvironments to soil factors under virus infection.

### 3.4 Associations of virus pathogen infection with ecological processes in phylogenetic bin assembly

The observed 1,642 ASVs were divided into 34 phylogenetic bins. Most microbial bins that exhibited increased deterministic processes had relatively low abundance (typically <5% relative abundance), yet demonstrated marked shifts in community assembly patterns. For example, in roots, the relative abundance of bin 17 in total bins was 2.44% in HR, and bin 17 in HR was 21.84% dominated by dispersal limitation, 63.75% dominated by homogeneous selection. The relative abundance of bin 17 in total bins was 5.09% in IR, and bin 17 in IR was 100% dominated by homogeneous selection. In the leaves, the relative abundance of bin 5 in total bins was 0.35% in HL, and bin 5 in HL was 47.74% dominated by homogeneous selection, 45.88% dominated by homogenizing dispersal. The relative abundance of bin 5 in total bins was 1.21% in IL, and bin 5 in IL was 100% dominated by homogeneous selection.

**Figure 6:**
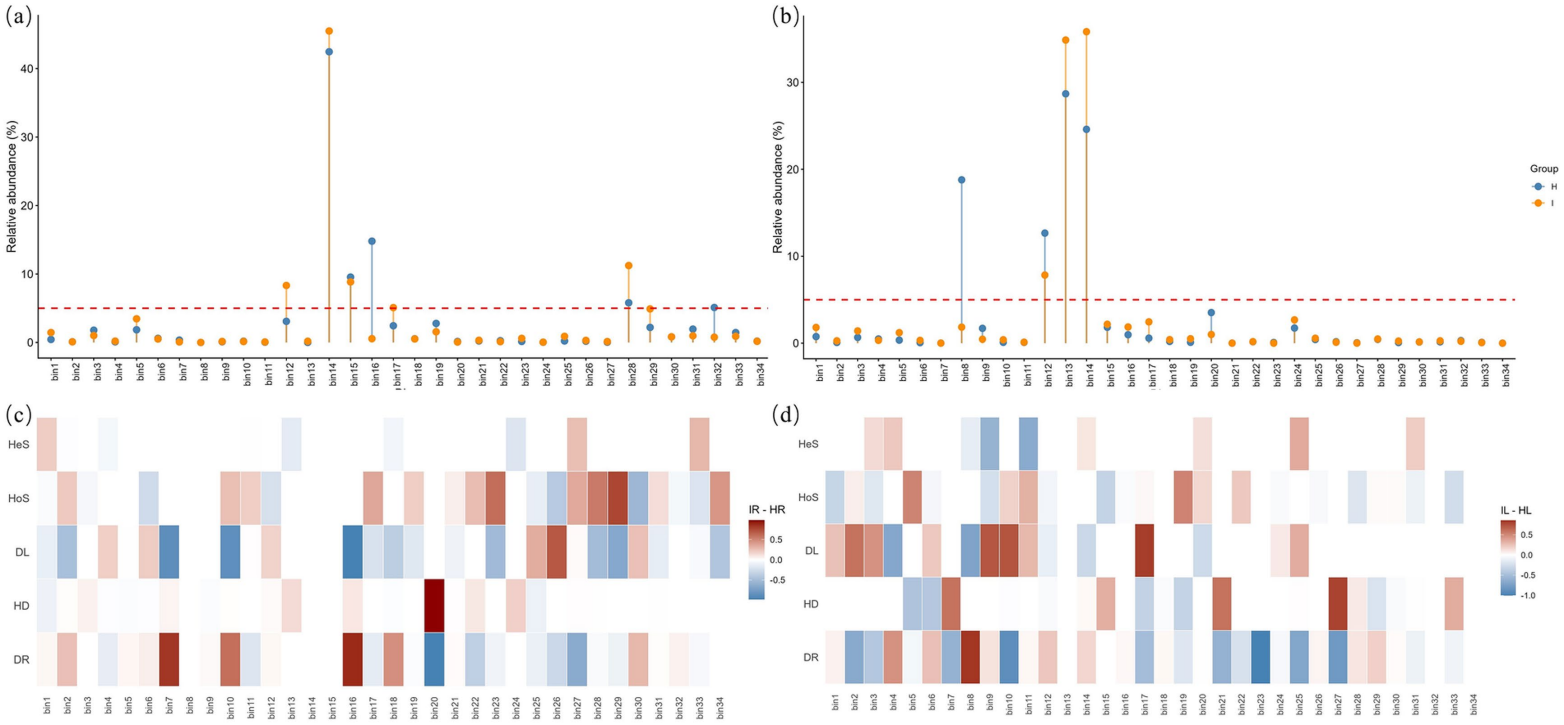
Panel **(a)** shows a lollipop plot comparing relative abundance percentages of bins between two groups, H and I, with H in blue and I in orange, and a red dashed 5% threshold line. Panel **(b)** presents a similar lollipop plot for another dataset, also comparing groups H and I. Panel **(c)** displays a heatmap representing IR minus HR values by bin and group, with a red-blue gradient key. Panel **(d)** features a heatmap for IL minus HL values by bin and group, using the same color gradient.

As shown in Figure 6, viral infection enhanced deterministic processes across multiple low-abundance bins in both roots and leaves. In roots, heterogeneous selection (HeS) was strengthened in bin 1, bin 27, and bin 33 (all with relative abundance <1.5%). In contrast, homogeneous selection (HoS) increased in bin 2, bin 10, bin 11, bin 17, bin 19, bin 22, bin 23, bin 27, bin 29, bin 31, and bin 34 (all with relative abundance <5%). A similar trend was observed in leaves, where HoS was reinforced in bin 2, bin 5, bin 10, bin 19, bin 20, and bin 22, and HeS was enhanced in bin 20 and bin 31, all with relative abundance below 4%.

Of the bins dominated by high-abundance species, a limited number underwent fundamental shifts in their community assembly processes alongside changes in relative abundance. Specifically, in roots, as Bin 28 transitioned from the healthy state (HR; relative abundance: 5.80%) to the infected state (IR; relative abundance: 11.23%), its assembly process shifted from being co-dominated by dispersal limitation (50.79%) and homogeneous selection (44.88%) to being entirely governed by homogeneous selection (100%). Conversely, in the leaves, as the relative abundance of Bin 14 increased from the healthy state (HL; 24.60%) to the infected state (IL; 35.83%), the deterministic forces in its assembly process weakened, while stochastic processes became more prominent. Specifically, homogeneous selection disappeared as a dominant force, replaced by heterogeneous selection (34.15%) and dispersal limitation (43.24%), alongside a substantial contribution from drift (17.04%). These results suggest that the ecological processes driving microbial taxa to dominance may differ fundamentally depending on the habitat (e.g., root vs. leaf).

PVY infection induced distinct shifts in the ecological assembly processes of key bacterial bins, with contrasting patterns between roots and leaves. Viral infection reduced stochastic processes while enhancing deterministic assembly in the roots. As illustrated in Figure 6, for the dominant species of bacteria, some bins increased the importance of the heterogeneous selection with the strengthening of PVY pathogen infection stress. For instance, *Nocardioides* (32.84% of the total abundance in bin 1) was 20.34% dominated by determinate processes in IR, while almost no heterogeneous selection in HR. *Sphingobacterium* (76.48% of the total abundance in bin 33) was 29.52% dominated by determinate processes in IR, while almost no heterogeneous selection in HR. In addition, some bins increased the importance of the homogeneous selection with the strengthening of virus stress. For example, *Delftia* (15.81% of the total abundance in bin 17) was 100% dominated by determinate processes in IR, while almost 63.75% of homogeneous selection in HR. *Clostridium* (96.77% of the total abundance in bin 28) was 100% dominated by determinate processes in IR, while almost 44.88% of homogeneous selection in HR. On the contrary, some bins decreased the importance of homogeneous selection with the increase of virus stress. Such as the proportion of *Flavobacterium* (99.11% of the total abundance in bin 30) has decreased by 54.35% in HR. While some bins increased the importance of the dispersal limitation with the strengthening of virus stress. The proportion of *Acinetobacter* (4.42% of the total abundance in bin 12) of dispersal limitation was increased by 17.79% in HR. The proportion of *Flavobacterium* (99.11% of the total abundance in bin 30) increased by 25.24% in HR. In leaves, virus stress changed the process of DR obviously. Within the leaves, the primary shift occurred within the stochastic spectrum itself, characterized by a notable decrease in dispersal limitation (DL) and a concurrent increase in drift (DR). For example, the proportion of *Methylobacterium* (100% of the total abundance in bin 8) of the DR process has increased 85.52% in IL, while the DL process has decreased 72.09%. The proportion of *Acinetobacter* (4.42% of the total abundance in bin 12) of the DR process has increased 23.35%, while the DL process has decreased 12.36%. The proportion of *Pantoea* (5.75% of the total abundance in bin 14) of the DR process has increased 17.04% in IL, while the DL process has decreased 30.18%. Minor transitions between drift (DR) and homogeneous selection (HD) were also observed. For instance, the proportion of *Lachnoclostridium* (59.00% of the total abundance in bin 29) of the DR process has increased 19.71% in IL, while the HD process has decreased 21.65%.

## 4 Discussion

This study investigated the effect of PVY pathogen infection on plant endophytic community diversity and assembly. By profiling bacterial communities in different plant compartments including roots and leaves of healthy and PVY pathogen infection plants. We found that PVY infection may induce a trend toward greater microbial evenness, but the observed changes fall within the range of natural variation and require further validation with larger sample sizes. More importantly, our results provide evidence that PVY pathogen infection enhances deterministic process in plant endophytic communities.

In healthy plants, soil properties showed little influence on endophytic assembly (only weak correlations with root stochastic processes), indicating that a healthy plant buffers its aboveground microbiome from direct soil effects and maintains stability—a key feature of soil–plant biological health. PVY infection broke this buffering. Soil factors began to significantly regulate deterministic assembly in both leaves and roots, creating a dual challenge: weakened plant control and increased soil forcing.

To understand the divergent responses of root and leaf endophytic communities to PVY stress, we consider the distinct selective environments of the two organs. Roots are directly connected to the soil microbial pool via the rhizosphere and are also exposed to systemic viral signals transported through the phloem. PVY infection alters root exudation patterns and cell wall integrity, which imposes strong selective filtering on incoming microbes. This explains why deterministic processes (especially homogeneous selection) increased markedly in infected roots, driving the community toward greater similarity. In contrast, leaves are the primary site of PVY replication and symptom expression. The virus induces localized defense responses, including oxidative bursts and cell death, which create heterogeneous microhabitats. These stochastic pockets of resources and niches promote ecological drift and heterogeneous selection, as observed in our data. Specifically, in infected leaves, both heterogeneous (HeS) and homogeneous (HoS) selection occurred; available potassium promoted HeS, total nitrogen and total potassium promoted HoS, while pH negatively regulated HeS. This suggests that infected leaves can flexibly switch between a “convergent defense” (HoS) and a “divergent utilization” (HeS) strategy depending on soil nutrients – a compensatory response. In infected roots, all significant correlations pointed to HeS, which was negatively regulated by soil water content, organic matter, and available potassium, and positively only by pH. Roots thus adopted a predominantly single strategy (HeS), but its intensity was suppressed by favorable soil conditions (e.g., high organic matter or water content), which may alleviate root stress and allow more stochastic assembly—revealing an indirect contribution of soil health indicators to disease resistance.

Notably, the same soil factor (e.g., available potassium) showed opposite effects on HeS in roots versus leaves. The proposed mechanistic interpretation is as follows. In leaves, potassium acts as a signaling molecule that upregulates defense-related genes (e.g., pathogenesis-related proteins), creating a specific chemical filter that enhances heterogeneous selection among epiphytic and endophytic bacteria (Armengaud et al., 2004). Conversely, in roots, higher available potassium may improve overall nutrient status and reduce root stress, thereby weakening the selective pressure imposed by the virus and suppressing HeS (Wang et al., 2013). This opposing pattern demonstrates that root-leaf coordinated management is essential for restoring or maintaining soil–plant biological health. The stark contrast between healthy and infected plants implies that microbiome studies using only healthy plants cannot predict assembly rules under viral stress; therefore, plant health status must be treated as a core variable when assessing soil–plant biological health. Precise regulation of soil factors (pH, potassium form and dosage, organic matter) may offer a way to directionally alter endophytic assembly and indirectly enhance plant resistance or tolerance to viral diseases, providing a novel approach for biological health regulation through soil management.

Deciphering keystone taxa, such as biomarker taxa, and the correlations with host plants and pathogens is critical for harnessing the plant microbiome to improve plant growth and health (Trivedi et al., 2020). Although PVY infection led to a reduction in the number of observed ASVs, the increase in the Chao1 richness estimator suggests that viral infection may have facilitated the emergence of numerous rare taxa, which were not fully captured in our sequencing due to their low abundance. Furthermore, alpha diversity indexes (Shannon index and Simpson index) increased slightly but not significantly. This suggests that while the virus eliminated some susceptible species, it also created opportunities for previously rare taxa to flourish, leading to a community restructured with greater uniformity in species abundances. Viral infection acts as a strong environmental filter that uniformly reduces species richness and, more importantly, reorders species’ relative abundances. Because the filtering outcome is influenced by subtle differences among individual hosts, community restructuring diverges across hosts, ultimately increasing compositional heterogeneity within the infected group.

Previous studies have shown that PVY pathogen infection causes a vast reprogramming of the host cell that results in cytological, biochemical and physiological changes (Baebler et al., 2020). Pathogen infection can shape the rhizosphere microbial communities and specifically accumulate a group of beneficial microbes (Yin et al., 2021). Our study extends this knowledge by showing that such reprogramming extends to the assembly rules of the associated microbial communities. Several potential beneficial bacteria, including *Streptomyces*, *Pantoea*, and *Delftia*, were significantly enriched in pathogen-infected plants and identified as biomarker taxa in the endophytic community. Notably, *Pantoea* and *Delftia* were found to be the dominant genera in key microbial bins (Bin 14 and Bin 17, respectively), underscoring their potential ecological significance within the community structure. Previous studies have revealed that many members of the *Pantoea* and *Streptomyces* genera colonize different plant compartments and play a vital role in modulating host performance, especially in plant pathogen suppression (Liu et al., 2020; Trivedi et al., 2020). Here, we further demonstrate that these beneficial taxa are not simply enriched but also undergo fundamental shifts in their ecological assembly processes. For example, *Delftia* in bin 17 shifted from partial homogeneous selection in healthy roots to complete homogeneous selection in infected roots, indicating strong deterministic recruitment by the stressed host.

Some studies have reported the plant growth-promoting effects of *Pantoea* and *Streptomyces* strains For example, *Pantoea* sp. 1.19 was isolated from the rhizosphere of rice plants from both the rhizosphere and as rice endophytes, and it can be used as a plant growth-promoting bacterium (Megias et al., 2017). *Pantoea* species have indeed shown that they have many beneficial traits that could be used in rice farming systems such as combating rice plant pathogens and promoting growth and fitness of the rice (Sun et al., 2020). *Streptomyces* is well known for excreting antibiotic compounds and can protect plants from pathogens (Lee et al., 2021). In this study, the host plant can selectively regulate they abundance of some species under virus pathogen stress. Under virus stress conditions, a plant can attract distant beneficial microbes by actively releasing nonvolatile root exudates, such as amino acids, nucleotides, and long-chain organic acids (Yuan et al., 2018), or by actively emitting blends of volatile organic compounds (Schulz-Bohm et al., 2018).

In the present study, we demonstrate that PVY infection acts as a strong environmental stressor that reshapes the internal bacterial communities of plants by enhancing deterministic selection processes, driving them toward greater similarity and specialization. This shift in community assembly may pose potential risks to tobacco and associated ecosystems, as it reduces the buffering capacity typically provided by stochastic community assembly. Understanding such virus-induced microbial changes is therefore critical for commercial crop production (Paluchowska et al., 2024). Therefore, it is urgent to study the response of microbial communities to virus stress. The processes and mechanism of community assembly and its relationship with community function are core issues of ecology (Ning et al., 2020; Stegen et al., 2013; Woodcock et al., 2007). While how viruses affect the assembly of bacterial microbial communities remained unclear. By examining the assembly process of bacterial communities by Phylogenetic-bin-based null model analysis (iCAMP), the changes in the relative importance of various processes affecting microbial communities were quantified. This study found that in the roots and leaves, the deterministic process increased at the virus stress, then only certain species adapted to these conditions were able to survive and reproduce, The root episphere microbiomes were determined by both host selection and soil characteristics(Chen et al., 2018; Xu et al., 2021). Through iCAMP, we further explored the different assembly mechanisms of bacterial communities, which could further explain the contribution of virus stress to the construction of microbial communities. As we know the plant exerts on these populations a filtering selection through the secretion of root exudates and the production of specific organic matter (Trivedi et al., 2020), Our iCAMP analysis quantified, for the first time in the context of a viral pathogen, the relative contributions of different ecological processes. In roots, homogeneous selection increased by 16% and dispersal limitation decreased by 17%, indicating that viral stress not only makes microbial communities more similar but also facilitates microbial movement and establishment—possibly through increased root porosity or altered exudation. In leaves, drift increased by 9% and dispersal limitation decreased by 13%, which may be explained by the high transpiration stream and stomatal density in leaves, increasing the probability of microbial immigration and successful establishment (Li L. et al., 2024).

Notably, statistical comparisons of nine soil physicochemical properties between healthy and infected groups revealed no significant differences (*p* > 0.05; Supplementary Table S1), ruling out soil heterogeneity as a confounding factor. We acknowledge that the relatively small sample size (six replicates per group) may limit the statistical power to detect subtle effects and precludes strong causal inference. Our conclusions should be interpreted as associations rather than definitive causal relationships. Future studies with larger sample sizes, ideally combined with longitudinal sampling and molecular confirmation of infection, are needed to validate and extend our findings.

## 5 Conclusion

Our study emphasized that PVY pathogen infection enhances deterministic processes in the assembly of the endophyte community in roots and leaves, with distinct patterns between the two organs. In roots, viral infection significantly increased both homogeneous (HoS) and heterogeneous (HeS) selection while markedly reducing the influence of dispersal limitation (DL), indicating that the input of endophytes from the soil microbial pool into roots is restructured under viral stress toward more deterministic and less spatially constrained assembly. In leaves, the assembly process was primarily reorganized within the stochastic spectrum: ecological drift (DR) increased significantly, while the decrease in dispersal limitation (DL) was not significant. The deterministic processes (HeS and HoS) and homogeneous dispersal (HD) also showed non-significant increasing trends. These contrasting patterns reflect the different selective environments: roots are directly influenced by soil and phloem-mediated systemic signals, whereas leaves experience localized defense responses and higher stochastic immigration.

Further, we found that the response of key microbes to viral stress impacted the overall community assembly model, such as the genus of *Delftia* and *Pantoea*. These beneficial taxa shifted toward more deterministic assembly under infection, suggesting that the host actively recruits them under disease pressure. From the perspective of soil–plant biological health, plant health status determines how soil physicochemical properties regulate endophytic assembly processes: healthy plants buffer aboveground microbiomes from direct soil effects, whereas viral infection disrupts this buffering capacity, leading to opposite regulatory responses of roots and leaves to the same soil factors (e.g., pH and potassium). Therefore, restoring or maintaining soil–plant biological health requires coordinated root-leaf soil management strategies, such as precise regulation of soil pH, organic matter content, and potassium form and application rate. This study significantly advances our understanding of the mechanisms underlying plant microbiome assembly under viral stress, and provides a theoretical and practical basis for enhancing crop resistance to viral diseases and promoting sustainable agricultural development through optimized soil biological health management.

## Data Availability

The datasets presented in this study can be found in online repositories. The names of the repository/repositories and accession number(s) can be found in the article/Supplementary material.
