# Supplementary material for: Viral pathogen infection drives deterministic assembly of plant endophytic microbiomes: soil properties as divergent drivers and low-abundance taxa as primary responders
Source: Front Microbiol. 2026 Jun 24;17:1866391. doi: 10.3389/fmicb.2026.1866391 (PMC13342043; doi:10.3389/fmicb.2026.1866391)
Supplement: Supplementary file 1 [file Table_1.DOCX]

**Supplementary Table S1.** Comparison of soil physicochemical properties between healthy and PVY‑infected groups. Data are presented as mean ± standard deviation (SD). Statistical tests were selected based on data distribution: if both groups passed the Shapiro‑Wilk normality test (p > 0.05) and the F‑test for equality of variances (p > 0.05), a standard t‑test (equal variance) was used; if normality held but variances were unequal, Welch’s t‑test was applied; if either group failed normality, the Wilcoxon rank sum test was used. Significance level was set at p < 0.05.

| Parameter | Healthy groups | PVY-infected groups | p-value |
| --- | --- | --- | --- |
| Water (%) | 13.87 ± 2.02 | 13.80 ± 2.39 | 0.96 |
| pH | 5.85 ± 0.55 | 6.08 ± 0.35 | 0.40 |
| OM (g/kg) | 21.25 ± 6.16 | 23.13 ± 2.99 | 0.52 |
| TN (g/kg) | 1.43 ± 0.55 | 1.62 ± 0.23 | 0.47 |
| AN (mg/kg) | 95.22 ± 33.50 | 99.43 ± 8.48 | 0.78 |
| AP (mg/kg) | 74.83 ± 49.96 | 121.83 ± 47.20 | 0.13 |
| AK (mg/kg) | 933.33 ± 560.73 | 1004.17 ± 305.54 | 0.79 |
| TP (g/kg) | 0.89 ± 0.34 | 1.15 ± 0.38 | 0.17 |
| TK (g/kg) | 13.40 ± 1.28 | 15.37 ± 4.11 | 0.52 |


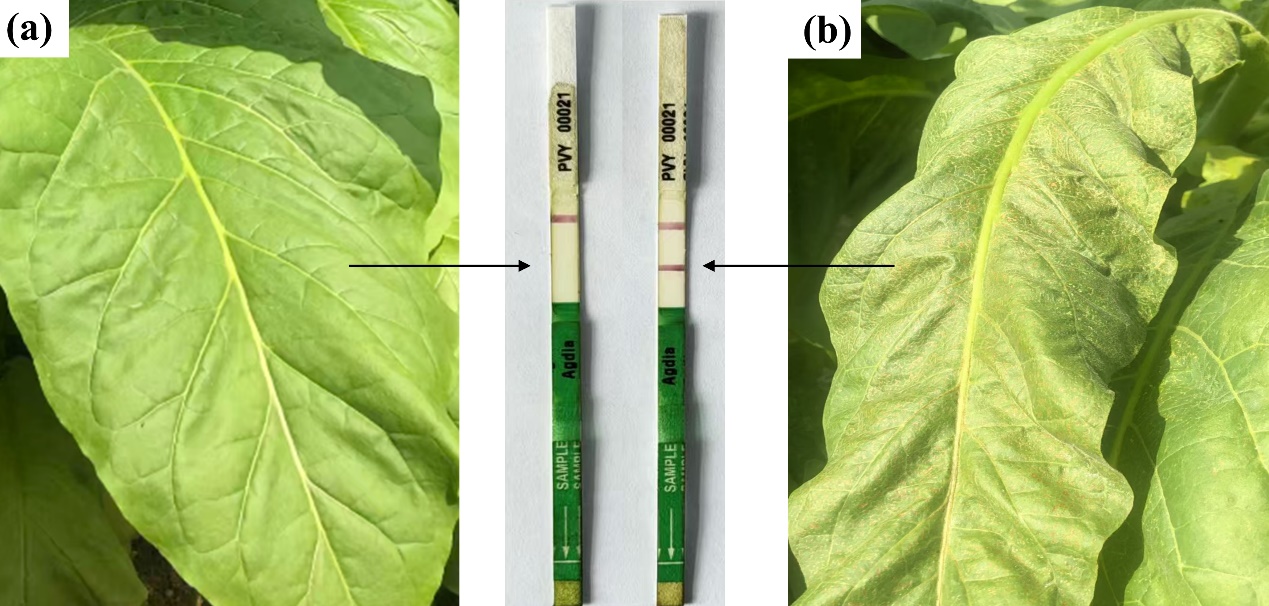


**Supplementary Figure S1.** PVY visual strip detection results. (a) Healthy tobacco sample: only the control line appears. (b) PVY-infected tobacco sample: both the control line and the test line appear. The intensity of the test line correlates with viral load.
